# Overrepresentation of IL-10-Expressing B Cells Suppresses Cytotoxic CD4^+^ T Cell Activity in HBV-Induced Hepatocellular Carcinoma

**DOI:** 10.1371/journal.pone.0154815

**Published:** 2016-05-02

**Authors:** Hongwei Xue, Fuhuang Lin, Hongwu Tan, Zun-Qiang Zhu, Zhang-Yun Zhang, Ludong Zhao

**Affiliations:** 1 Department of Oncology, the Affiliated Hospital of Qingdao University, Qingdao, 266003, Shandong, China; 2 Department of Interventional Radiology, Hainan Province People’s Hospital, Haikou, Hainan, 570311, China; 3 Department of Hepatobiliary Surgery, Linyi People’s Hospital, Shandong, 276000, China; 4 Department of Medicine, Sixth People'‘s Hospital, Shanghai Jiaotong University, Shanghai, China; University of Cape Town, SOUTH AFRICA

## Abstract

Hepatocellular carcinoma (HCC) is a common cancer with poor prognosis and low five-year survival rate. A strong and effective CD4^+^ T cell-mediated cytotoxicity was associated with better survival and low recurrence rate in HCC, but the regulatory mechanism that controls CD4^+^ T cell cytotoxicity in HCC patients is not fully examined. Given that IL-10-expressing B cells could suppress the inflammation of cytotoxic CD8^+^ T cells, T helper 1 (Th1) cells and Th17 cells, while promoting regulatory T (Treg) cell differentiation, we examined the role of IL-10-expressing B cells in HBV-related HCC patients. We found that compared to healthy controls, HCC patients exhibited significantly higher frequencies of IL-10-expressing B cells, which were negatively correlated with the frequencies of granzyme A, granzyme B, and perforin expressing CD4^+^ T cells. Surface molecule Tim-1 was preferentially expressed on IL-10-expressing B cells. Therefore, we separated total B cells into Tim-1^+^ and Tim-1^-^ B cells. CD4^+^ T cells incubated with Tim-1^+^ B cells exhibited significantly reduced levels of granzyme A, granzyme B and perforin expression, compared to the CD4^+^ T cells incubated with Tim-1^-^ B cells. Antagonizing IL-10 in culture rescued CD4^+^ T cell cytotoxicity. Compared to that in peripheral blood, the level of IL-10-expressing B cells were further upregulated in resected tumor, while the level of CD4^+^ cytotoxic T cells was downregulated. The negative correlations between IL-10-expressing B cells and CD4^+^ cytotoxic T cells were also observed in tumor-infiltrating cells. Together, our data revealed an additional antitumor mechanism mediated by IL-10-expressing B cells.

## Introduction

Hepatocellular carcinoma (HCC) is one of the most common cancers in Asia, and can be induced by many risk factors, such as alcoholism, hepatitis B virus (HBV) and hepatitis C virus (HCV) infections, and liver cirrhosis [1–3]. In China, the most frequent cause of HCC is endemic childhood HBV infection [4,5]. Serum HBV DNA level is directly correlated with increased risk of HCC development [4]. A strong and effective HBV-specific CD8^+^ T cell-mediated cytotoxicity is thought to play a crucial role in controlling cancer development as well as controlling HBV infection [6]. Recently, CD4^+^ T cell-mediated cytotoxicity is being increasingly recognized for its role in virus control and antitumor immunity [7,8]. CD4^+^ cytotoxic T cells are defined by their characteristic granzyme and perforin expression in response to MHC class II-restricted antigens [9], and have been discovered chronic virus infections, autoimmune diseases, and circulatory tumors [8,10,11]. In HCC, circulating and tumor-infiltrating CD4^+^ cytotoxic T cells are increased in early stages of HCC but are decreased in advanced stages; loss of CD4^+^ cytotoxic T cells is significantly correlated with high mortality rate and reduces survival time of HCC patients [12]. These data indicate an active role of CD4^+^ T cell-mediated cytotoxicity in antitumor immune responses in HCC, and suggest the existence of a regulatory mechanism of inhibiting cytotoxic CD4^+^ T cells.

The regulatory B (Breg) cells have been shown to prevent the induction of autoimmune responses and suppress excessive inflammation in autoimmune diseases by promoting regulatory T (Treg) cell differentiation and suppressing T helper 1 (Th1) and Th17 inflammation. In virus infection, they could also inhibit virus-specific CD8+ T cell responses and promote virus persistence [13]. In chronic HBV infection, the frequency of IL-10-expressing Bregs is upregulated, and could suppress HBV-specific CD8^+^ T cell responses through the production of inhibitory cytokine IL-10. IL-10 expressing Bregs is also associated temporally with hepatic flares [14]. It has been reported that B cell-deficient mice exhibit enhanced antitumor immunity, possibly due to the reduction of IL-10 produced by B cells when the CD40 expressed on B cells interacts with CD40L expressed by tumor cells [15]. Collectively, these studies suggest that Breg cells and B cell-mediated IL-10 production might play an inhibitory role in HCC. Also, B cells express MHC class II molecules and are capable of presenting antigen to CD4^+^ cytotoxic T cells, which raises the question of whether IL-10-producing Breg cells could mediate the suppression of CD4^+^ cytotoxic T cells in late stage HCC.

To answer that question, we examined the frequencies of IL-10-producing B cells and granzyme- and perforin-expressing CD4^+^ T cells in HCC patients. We found that the frequency of IL-10-producing B cells was negatively correlated with that of granzyme- and perforin-expressing CD4^+^ T cells. Incubation with IL-10-expressing B cells significantly reduced the granzyme and perforin expression by CD4^+^ T cells. Moreover, these effects were further elevated in HCC tumor resections. Together, we discovered a mechanism through which the CD4^+^ T cell-mediated cytotoxicity was regulated.

## Materials and Methods

### Ethical statement

All subjects were recruited under a protocol approved by the ethics committees at Linyi People’s Hospital and Sixth People's Hospital. Written consent was obtained from all participants.

### Study subjects

Surgically removed tumor samples, as well as peripheral blood samples prior to surgery, were obtained from all HCC patients. HCC was diagnosed according to the American Association for the Study of Liver Diseases (AASLD) guidelines [16]. Staging was based on the TNM classification system. Patients with concurrent infections, autoimmune diseases or alcoholic liver diseases were excluded.

### Sample Preparation

Peripheral blood mononuclear cells (PBMCs) were isolated from blood by the standard Ficoll-Hypaque (GE Healthcare) procedure and frozen immediately at -80°C until use. Freshly resected tumor were first cut into small cubes, minced and digested in 50 mL Hank’s balanced salt solution supplemented with 40 mg collagenase, 4 mg DNase I and 100 U hyaluronidase (Sigma) for 1 h in 37°C with shaking, and filtered through a 100-μm strainer (Falcon) to obtain homogenized cell suspension. Tumor-infiltrating lymphocytes were then obtained by standard Ficoll-Hypaque procedure.

### Cell Isolation

B cells were first isolated using Human B cell Enrichment Kit (Stemcell) with greater than 97% purity. The separation of Tim-1^+^ and Tim-1^-^ B cells were then done by first staining purified B cells with APC-conjugated anti-human Tim-1 antibody, followed by using APC Selection Kit (Stemcell) to separate the Tim-1^+^ B cells (purity > 95%) from Tim-1^-^ B cells (Purity > 99.5%). CD4^+^ T cells were isolated using Human CD4 T cell Enrichment Kit (Stemcell).

### Incubation

All cells were incubated in RPMI 1640 with FBS, Pen Strep and L-glutamine (Invitrogen) in 37°C 5% CO_2_. For B cell-T cell coculture, 10^5^ Tim-1^+^ or Tim-1^-^ B cells were incubated with 10^5^ autologous CD4^+^ T cells per 200 μL culture media for 72 h, at 1:1 ratio, following previously established protocols [17,18]. Recombinant soluble IL-10 receptor (sIL-10R) (R&D systems) was added at 3 μg/mL. Staphylococcal enterotoxin B (SEB) was added at 1 μg/mL. Brefeldin A (Invitrogen) was added at 10 μg/mL 5 h before staining and flow cytometry.

### Flow Cytometry

Surface staining was done by incubating cells with different combinations of anti-human CD3-BV 650, CD4-BV 785, CD19-BV 605, Tim-1-APC monoclonal antibodies (BioLegend), and Violet Dead Cell stain (Invitrogen) for 30 min at 4°C. Cells were then washed and treated with Intracellular Fixation and Permeabilization Buffer set (eBioscience). Anti-human IL-10-PE, granzyme A-FITC, granzyme B-APC and/or perforin-PE monoclonal antibodies were then added, followed by 30 min 4°C incubation, after which cells were washed twice and fixed with 2% formaldehyde. Samples were acquired in BD Fortessa and analyzed in FlowJo.

### Statistical Analyses

Ordinary or paired ANOVA was used for comparisons between multiple groups and then Sidak’s test was used for pair-wise comparisons. Paired or unpaired *t* tests were used for comparisons of two groups. Pearson’s correlation was used for correlation analysis. All statistical analyses were done using Prism (GraphPad Software). P < 0.05 was considered significant.

## Results

### Clinical information of HCC patients and healthy controls

The clinical data of all participants are shown in Table 1. All HCC patients had been infected with HBV chronically for over 20 years, while the age-matched, sex-matched healthy controls were not infected with HBV as confirmed by the lack of HBV antigens and DNA. Peripheral blood samples from all study participants and resected tumor samples from HCC patients following surgery were examined.

**Table 1:** Characteristics of the study population.

	HCC (N = 15)	Control (N = 13)
Age (mean±SD)*	51.5 ± 11.4	50.2 ± 12.0
Gender (M/F)	15/0	13/0
HBsAg (+/-)	15/0	0/13
HBeAg (+/-)	6/9	0/13
HBcAg (+/-)	15/0	0/13
HBV DNA (+/-/ND)	11/4/0	0/0/13
Tumor Stage (II/III)	8/5	-

* Individual data points are provided in the supporting information (S1 Table).

### Identification of IL-10-expressing B cells and granzyme and perforin-expressing CD4^+^ T cells in healthy controls and HCC subjects

**Fig 1:**
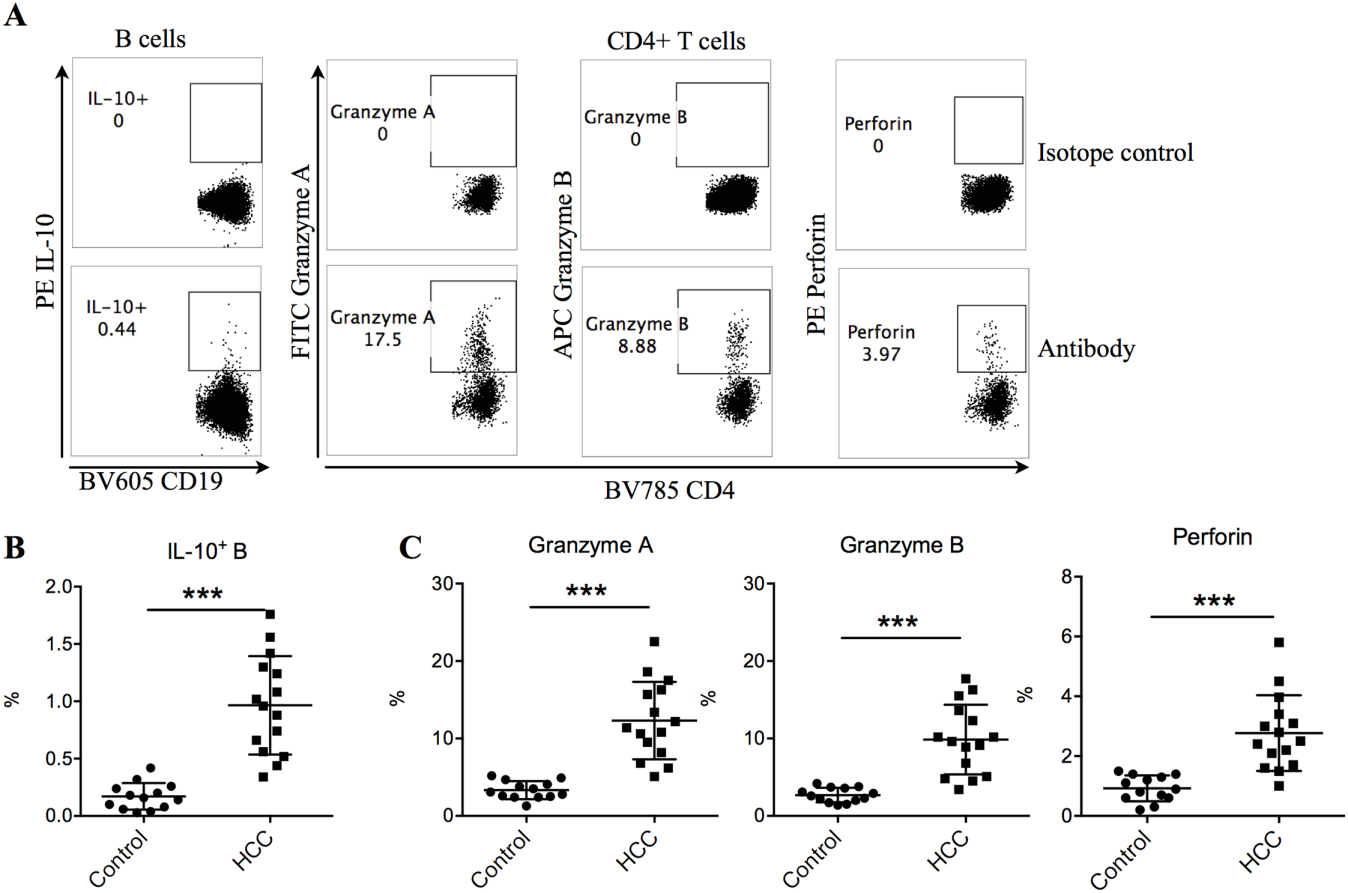
Significantly higher frequencies of IL-10-expressing B cells and granzyme A, granzyme B and perforin-expressing CD4+ T cells were observed in HCC patients than healthy controls. (A) Representative gating strategy of IL-10-expressing B cells and granzyme A, granzyme B and perforin-expressing CD4+ T cells in one HCC patient. B cells were gated as CD19+CD3- lymphocytes while CD4+ T cells were gated as CD19-CD3+CD4+ lymphocytes. (B) Frequencies of IL-10-expressing (IL-10+) B cells in total B cells in the peripheral blood of healthy controls and HCC patients. (C) Frequencies of granzyme A, granzyme B and perforin-expressing CD4+ T cells in total CD4+ T cells in the peripheral blood of healthy controls and HCC patients. Welch’s *t* test. Mean ± SD. *** *P* < 0.001.

Compared to healthy controls, we found that HCC subjects contained significantly higher levels of circulating IL-10-expressing B cells (Fig 1A and 1B). To examine the cytotoxic activity of CD4^+^ T cells, granzyme A-, granzyme B- and perforin-expressing CD4+ T cells in the peripheral blood mononuclear cells were identified as cytotoxic T cells, which were significantly elevated in HCC patients compared to healthy controls (Fig 1A and 1C). Overall, we observed significantly elevated frequencies of IL-10-producing B cells and cytotoxic CD4^+^ T cells in HCC patients.

### Frequencies of IL-10-expressing B cells were negatively correlated with frequencies of cytotoxic CD4^+^ T cells in HCC patients

**Fig 2:**
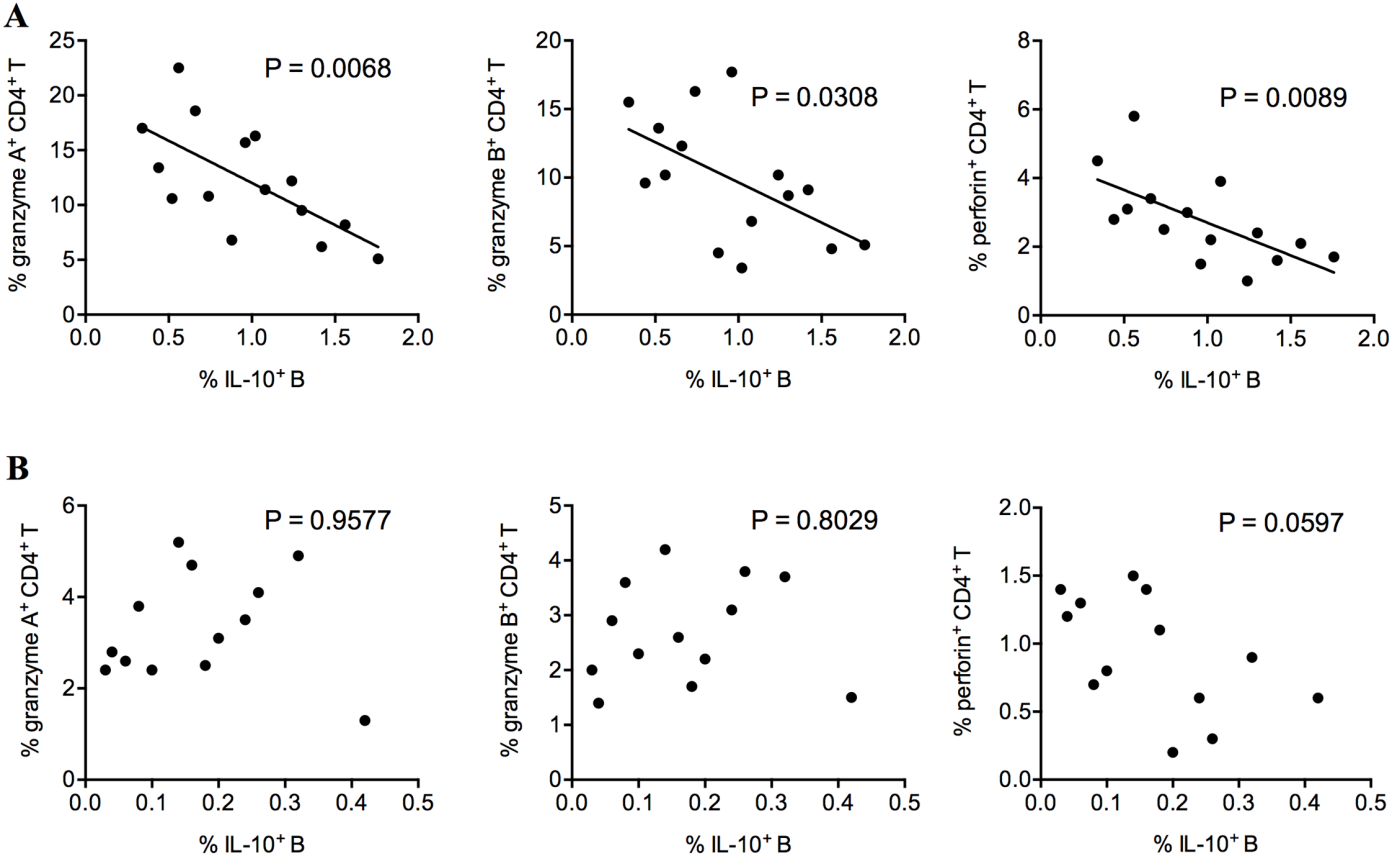
The frequencies of granzyme A, granzyme B and perforin-expressing CD4+ T cells were negatively correlated with the frequencies of IL-10-expressing B cells in the peripheral blood of HCC patients. (A) The correlations between the peripheral blood frequencies of IL-10-expressing B cells and granzyme A-, granzyme B- and perforin-expressing CD4+ T cells in HCC patients. (B) The correlations between the peripheral blood frequencies of IL-10-expressing B cells and granzyme A-, granzyme B- and perforin-expressing CD4+ T cells in healthy controls. Pearson correlation test. *P* < 0.05 was considered significant.

IL-10-expressing B cells were previously demonstrated to suppress CD8+ T cell inflammation in HIV and HBV infections, autoimmune diseases, and tumor [14,15,17,19]. Their role in suppressing CD4^+^ T cell cytotoxicity has not been examined, but their surface expression of MHC class II molecules potentially enables interaction with CD4^+^ cytotoxic T cells. Here, we observed that the frequencies of granzyme A, granzyme B, and perforin-expressing CD4+ T cells were negatively correlated with the frequencies of IL-10-expressing B cells in HCC patients (Fig 2A, S2 Table). Such correlations were not observed in healthy controls (Fig 2B).

### IL-10-expressing B cells suppressed CD4^+^ T cell cytotoxic activities *in vitro*

**Fig 3:**
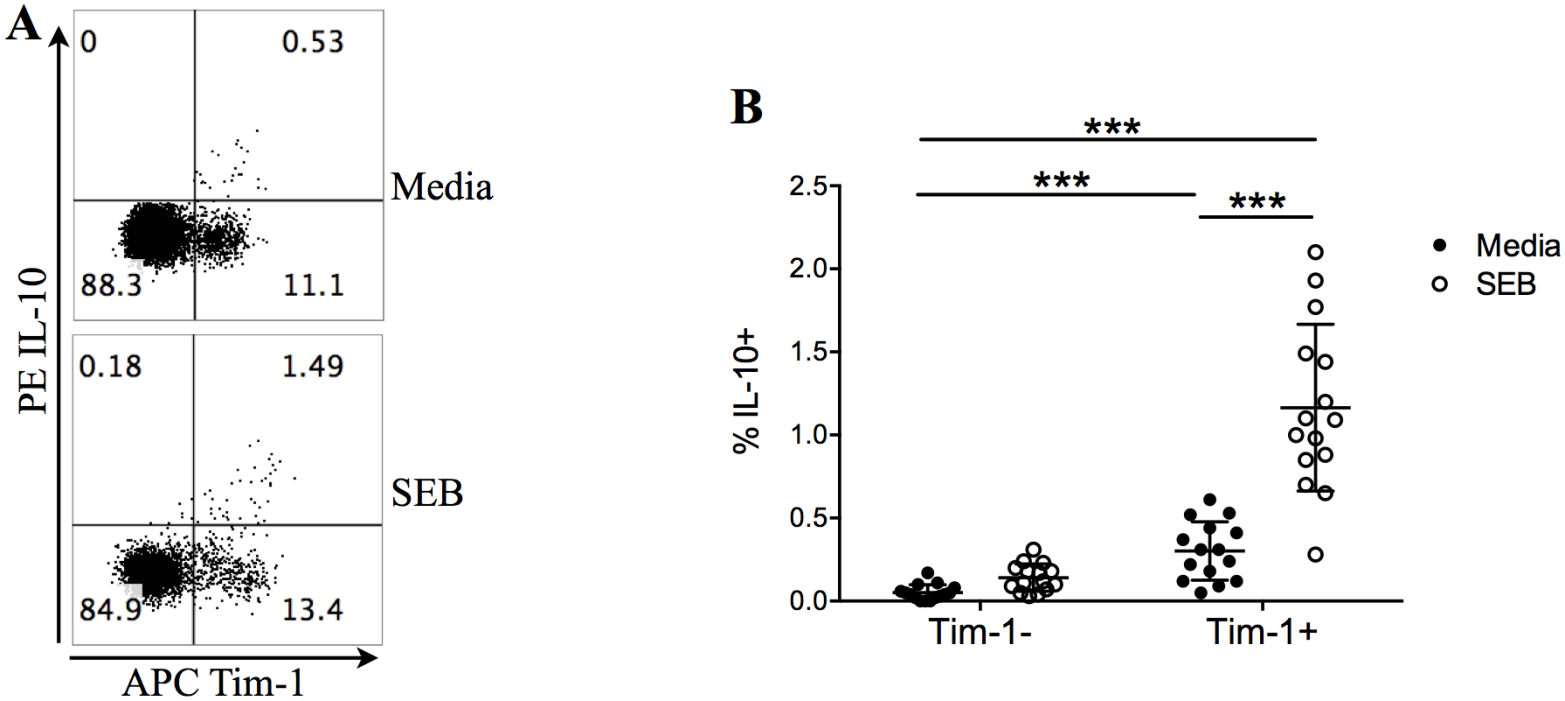
IL-10 is preferentially expressed by Tim-1^+^ B cells in HCC patients. (A) Representative IL-10 expression by Tim-1+ and Tim-1- B cells in one HCC patient, before or after stimulation with 1 μg/mL SEB for 72 h. (B) IL-10 expression by Tim-1^+^ vs. Tim-1^-^ B cells in all HCC patients, before or after SEB stimulation. Two-way ANOVA followed by Sidak’s multiple comparisons test. Mean ± SD. *** *P* < 0.001.

**Fig 4:**
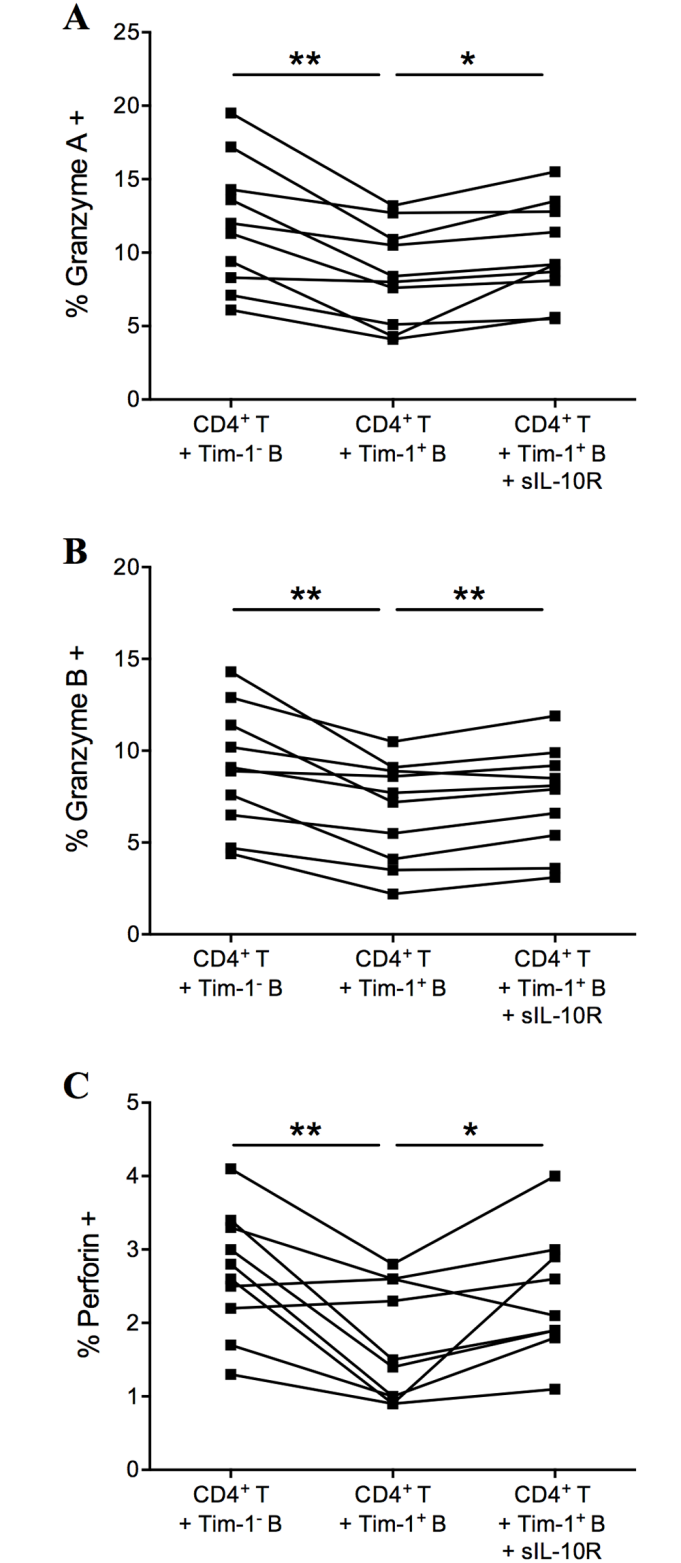
Tim-1+ B cells suppressed CD4+ T cell granzyme A, granzyme B, and perforin expression through IL-10-mediated mechanism. 105 purified Tim-1+ or Tim-1- B cells were incubated with 105 autologous purified CD4+ T cells with 1 μg/mL SEB for 72 h. 1 μg/mL sIL-10R was added in a subset of cultures to antagonize IL-10. The (A) granzyme A, (B) granzyme B, and (C) perforin expression by CD4+ T cells were then measured by flow cytometry. RM one-way ANOVA followed by Sidak’s multiple comparisons test. ** *P* < 0.01. * *P* < 0.05.

To examine whether IL-10 production by B cells could directly modulate CD4^+^ T cell cytotoxic response, we first examined surface markers that could be used to selectively deplete IL-10-expressing B cells. T cell immunoglobulin and mucin-domain containing 1 (Tim-1) expression is essential for the induction and maintenance of IL-10 in Bregs, and has been used to enrich or deplete IL-10-expressing B cells [17,20,21]. By separating B cells according to their Tim-1 expression, we found that Tim-1^+^ B cells expressed significantly higher IL-10 before and after SEB stimulation than Tim-1^-^ B cells (Fig 3A and 3B). Therefore, we used Tim-1 as an IL-10-expressing B cell marker, and separated whole B cells into Tim-1^-^ and Tim-1^+^ B cell fractions, which were then coincubated with autologous CD4+ T cells with SEB stimulation. We found that CD4^+^ T cells incubated with Tim-1^+^ B cells had significantly lower levels of granzyme A, granzyme B and perforin expression, than CD4^+^ T cells incubated with Tim-1^-^ B cells (Fig 4A to 4C). Antagonizing IL-10 by adding recombinant soluble IL-10 receptor (sIL-10R) in the Tim-1^+^ B cell culture has reverted this effect. Together, these data demonstrated that the Tim-1^+^ B cells could directly suppress CD4^+^ T cell cytotoxicity through IL-10-mediated mechanisms.

### IL-10-expressing B cells were further upregulated in resected tumor with a significant reduction of cytotoxic CD4+ T cells

**Fig 5:**
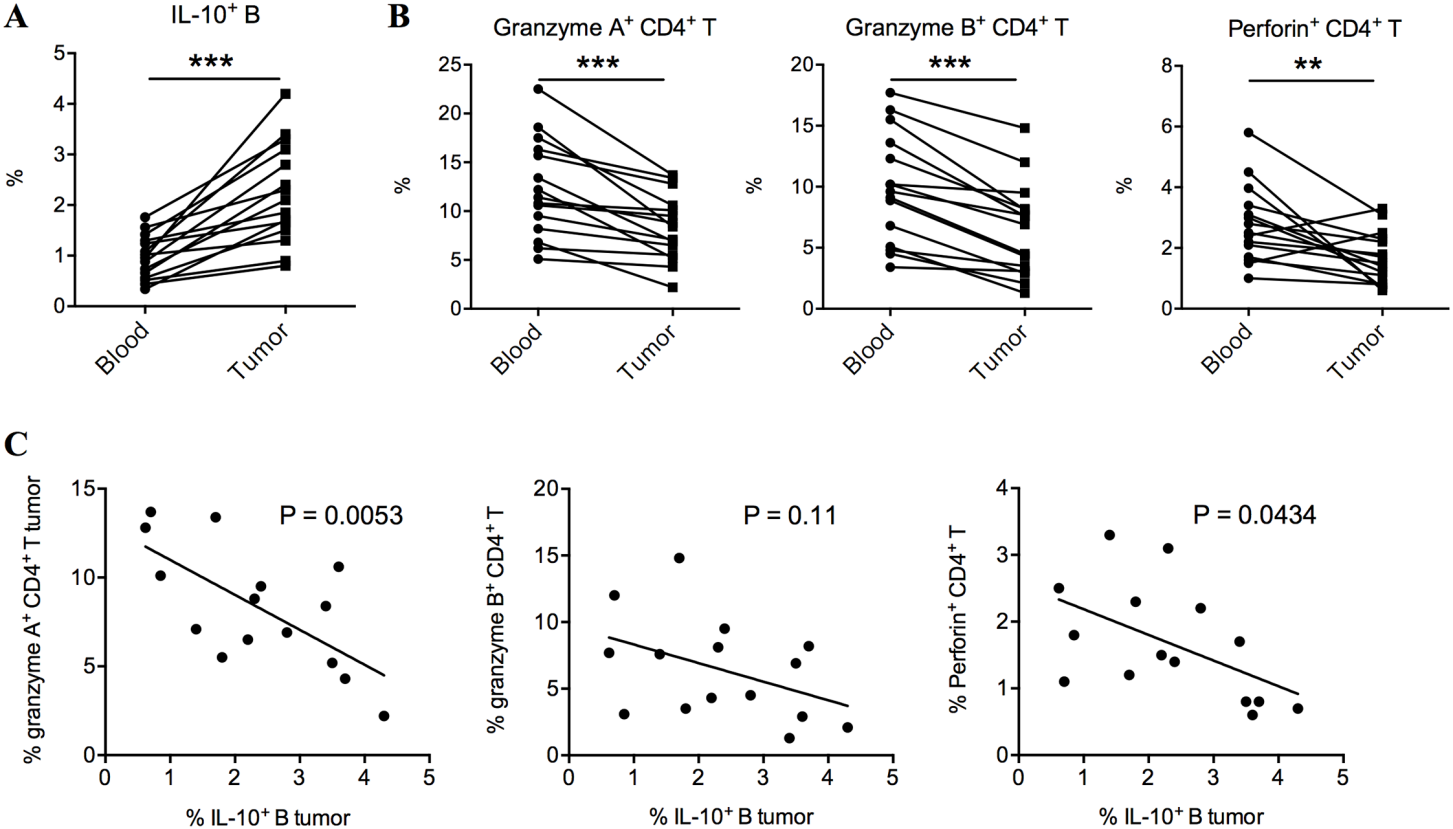
Compared to peripheral blood, tumor-infiltrating IL-10-expressing B cells were further upregulated and CD4+ cytotoxic T cells were downregulated. (A) The frequencies of IL-10-expressing B cells in the peripheral blood and resected tumor of the same subject. (B) The frequencies of granzyme A, granzyme B and perforin-expressing in the peripheral blood ans resected tumors of the same subject. Paired *t* test. *** *P* < 0.001. ** *P* < 0.01. (C) The correlations between the frequencies of tumor-infiltrating IL-10-expressing B cells and tumor-infiltrating granzyme A, granzyme B and perforin-expressing CD4+ T cells in HCC patients. Pearson correlation test. *P* < 0.05 is considered significant.

The frequencies of tumor-infiltrating CD4^+^ T cells were previously associated with longer disease-free survival periods and better prognosis [12]. We examined the correlation between IL-10-expressing B cells and CD4^+^ T cells in the resected tumor. We found that a significantly higher frequencies of IL-10-expressing B cells and significantly lower frequencies of CD4^+^ cytotoxic T cells in the tumor, compared to their counterparts in peripheral blood (Fig 5A and 5B). The frequencies of tumor-infiltrating IL-10-expressing B cells and the frequencies of granzyme A and perforin-expressing CD4^+^ T cells were negatively correlated (Fig 5C).

## Discussion

B cell-mediated IL-10 expression is known to suppress CD4^+^ T cell proinflammatory cytokine expression, such as TNF-α, IFN-γ, and IL-17, inhibit CD8+ T cell cytotoxicity, and promote Treg cell differentiation. Also, it enables virus persistence in chronic infections and inhibit antitumor responses [15,22]. In chronic HBV infections, CD19^+^CD24^hi^CD38^hi^ B cells that could express IL-10 are upregulated and are shown to suppress HBV-specific CD8^+^ T cells [14]. However, their effect on CD4^+^ cytotoxic T cells, a cell type that is associated with better prognosis in HBV-related HCC, is not examined. Here, we found that compared to healthy controls, HCC patients exhibited significantly higher frequencies IL-10-expressing B cells, which were negatively correlated with the frequencies of granzyme A-, granzyme B-, and perforin-expressing CD4^+^ T cells. Surface molecule Tim-1 was preferentially expressed on IL-10-expressing B cells. CD4^+^ T cells incubated with Tim-1^+^ B cells exhibited significantly reduced levels of granzyme A, granzyme B and perforin expression, compared to the CD4^+^ T cells incubated with Tim-1^-^ B cells. Antagonizing IL-10 in culture rescued CD4^+^ T cell cytotoxicity. Compared to that in peripheral blood, the level of IL-10-expressing B cells was further upregulated in resected tumor, while the level of CD4^+^ cytotoxic T cells was downregulated. Negative correlations between the frequencies of IL-10-expressing B cells and the frequencies of granzyme A- and perforin-expressing CD4^+^ T cells were also observed in tumor-infiltrating cells. Together, these data demonstrated an additional mechanism through which IL-10-expressing B cells could suppress antitumor immunity.

Currently, it is unclear how the frequency of IL-10-expressing B cells is upregulated in the peripheral blood of HBV-related HCC patients and is further increased in tumor resections. The liver microenvironment is in general considered refractory to immune activation due to the presence of many regulatory mechanisms, such as antigen-presentation by inhibitory liver-resident Kupffer cells and sinosoidal endothelial cells and hyporesponsiveness toward pro-inflammatory stimuli such as LPS [23]. Increase of IL-10-expressing B cells could contribute to the immune inhibition in the liver microenvironment. On the other hand, a diverse range of metabolites, wastes and toxic compounds are found in the liver, which could interact with Toll-like receptors (TLRs) on the B cells. Previously, TLR-2 and TLR-9 agonists were shown to increase IL-10 production in B cells [17,24]. Here, it is unknown whether IL-10-expressing B cells could be induced intratumorally, or preferentially trafficked to the tumors. This should be investigated in detail because B cells are composed of multiple subsets, some of which are important components in tumor lymphoid structures and can act as antigen-presenting cells to T cells [25]. Recently, another study by Garnelo et al. showed that the density of tumor-infiltrating B cells in HCC correlated with the frequency and activation of tumor T cell and natural killer (NK) cells; depletion of B cells in mice significantly enhanced the growth of murine hepatoma cell lines and reduced CD4^+^ T cell activation, including granzyme B production [24]. Studies in breast and ovarian cancers also showed that increased tumor-infiltrating CD20^+^ B cells were associated with favorable outcomes [25,26]. These studies and ours therefore highlighted the importance of investigating the underlying mechanisms that control the balance between intratumoral proinflamatory and antiinflammatory B cell responses.

Others and we have observed an increase in CD4^+^ cytotoxic T cells in the peripheral blood of HCC patients compared to healthy individuals [12], but the underlying mechanism is unclear. Previous examinations have shown that the frequency of CD4^+^ cytotoxic T cells is partially increased in a subset of patients with chronic HBV infection, but not to the extent in HCC patients [12,27]. Studies have also shown that CD4^+^ T cells produce perforin only in the absence of CD8^+^ T cell cytotoxicity [28]. And they are found in chronic viral infections (such as HIV and LCMV) that possess the ability to suppress MHC class I antigen presentation [7]. These studies suggest that the suppression of CD8^+^ T cell- and NK cell-mediated cytotoxicity in HCC might have induced CD4^+^ T cell cytotoxicity by feedback mechanisms [12,29–31]. On the other hand, given that CD8^+^ T cell cytotoxicity mediates liver damage in HBV-infected individuals [32], the potential pathogenic roles of the increase in CD4^+^ cytotoxic T cells should be examined. Whether IL-10-producing B cells protect the liver from damage also require further examinations.

Tim-1, the surface marker used in this study to enrich for IL-10-expressing B cells, is also expressed on CD4^+^ T cells and has been shown to increase the expression of IL-4, but not IFN-γ[33]. In mice, Tim-1 is induced on B cells in the germinal center, but Tim-1 knockout did not abrogate germinal center development or antibody responses [34]. Ligation of Tim-1 with low affinity Tim-1 antibody RMT-10 could directly induce IL-10-producing B cells in mice [21]. These studies suggest that Tim-1 preferentially mediates regulatory responses. Whether Tim-1 could be used as a drug target to rescue the suppressed tumor immunity would require further studies.

## Supporting Information

S1 Table Age of the participants. (XLSX)

S2 Table Raw data of the analyses. (PZF)
